# Maternal serum concentration of anti-Müllerian hormone is a better predictor than basal follicle stimulating hormone of successful blastocysts development during IVF treatment

**DOI:** 10.1371/journal.pone.0239779

**Published:** 2020-10-12

**Authors:** Sheela Sadruddin, Brian Barnett, Lowell Ku, Dara Havemann, Sara Mucowski, Richard Herrington, Warren Burggren

**Affiliations:** 1 Developmental Integrative Biology Research Group Department of Biological Sciences University of North Texas Denton, Denton, Texas, United States of America; 2 Dallas IVF Frisco, Frisco, Texas, United States of America; 3 Research Data Science University of North Texas Denton, Denton, Texas, United States of America; China Agricultural University, CHINA

## Abstract

**Background:**

The conditions of diminished ovarian reserve and primary ovarian insufficiency, characterized by poor fertility outcomes, currently comprise a major challenge in reproductive medicine, particularly *in vitro* fertilization. Currently in the IVF industry, blastocyst developmental success rate per treatment is routinely overlooked when a live birth results from treatment. Limited data are available on this significant and actionable variable of blastocyst development optimization, which contributes to improvement of treatment success Women with elevated basal FSH concentration are reported to still achieve reasonable pregnancy rates, although only a few studies report correlations with blastocysts development. Diagnostic values of AMH/basal FSH concentrations can be useful for determining the optimal stimulation protocol as well as identification of individuals who will not benefit from IVF due to poor prognosis. The objective of this study is to identify actionable clinical and culture characteristics of IVF treatment that influence blastocyst developmental rate, with the goal of acquiring optimal success.

**Methods and findings:**

A retrospective observational study was performed, based on 106 women undergoing IVF, regardless of prognosis, over a six-month period from January 1, 2015 to June 31, 2015. Rate of high-quality blastocyst production, which can be used for embryo transfer or vitrification, per normally fertilized oocyte, was evaluated. Treatment was determined successful when outcome was ≥ 40% high-quality blastocysts. The data were initially evaluated with the *Evtree* algorithm, a statistical computational analysis which is inspired by natural Darwinian evolution incorporating concepts such as mutation and natural selection (see Supplementary Material). The analysis processes all variables simultaneously against the outcome, aiming to maximize discrimination of each variable to then create a “branch” of the tree which can be used as a decision in treatment. The final model results in only those variables which are significant to outcomes. Generalized linear model (GLM) employing logistic regression and survival analysis with R software was used and the final fitting of the model was determined through the use of random forest and evolutionary tree algorithms. Individuals presenting with an [AMH] of >3.15 ng/ml and a good prognosis had a lower success per treatment (n = 11, 0% success) when total gonadotropin doses were greater than 3325 IU. Individuals that presented with an [AMH] of <1.78 ng/ml and a poor prognosis exhibited a greater success per treatment (n = 11, 80% success). AMH emerged as a superior indicator of blastocyst development compared to basal FSH. The accuracy of the prediction model, our statistical analysis using decision tree, evtree methodology is 86.5% in correctly predicting outcome based on the significant variables. The likelihood that the outcome with be incorrect of the model, or the error rate is 13.5%.

**Conclusions:**

[AMH] is a superior indicator of ovarian stimulation response and an actionable variable for stimulation dose management for optimizing blastocyst development in culture. Women whose [AMH] is ≥3.2 mg/ml, having a good prognosis, and developing >12 mature follicles result in <40% blastocysts when gonadotropin doses exceed 3325 IU per treatment. IVF treatments for poor responders that present with infertility due to diminished ovarian reserve, if managed appropriately, can produce more usable blastocyst per IVF treatment, thus increasing rate of blastocyst developmental success and ultimately increasing live birth rates. Future studies are needed to investigate the intra-follicular and the intra-cellular mechanisms that lead to the inverse relationship of blastocysts development and total gonadotropin doses in good responders in contrast to poor responders.

## Introduction

The conditions of diminished ovarian reserve (DOR) and primary ovarian insufficiency (POI) are characterized by poor fertility outcomes and currently present as a major challenges in reproductive medicine, particularly *in vitro* fertilization (IVF) [1, 2]. Women are diagnosed with DOR if they demonstrate at least one of the following: high basal follicle stimulating hormone (FSH) levels, above 6.5 mIU/ml, in early follicular phase, low antral follicle counts of <12 or Low anti-Mullerian hormone (AMH) of <1 ng/ml. A woman in her reproductive years is considered to have good prognosis for IVF when her basal follicle stimulating hormone (FSH) plasma concentration is between 3.1–7.9 mIU/ml and her anti-Müllerian hormone (AMH) plasma concentration is ≥1 ng/ml [2–4]. [AMH] is used to assess a woman’s remaining ovarian reserve as well predict the response to controlled ovarian hyperstimulation during IVF treatment [5–7]. Serum concentrations of FSH have been used in assessing women’s health for many years. [FSH] laboratory studies have been the standard for years and are routinely performed for IVF treatment patients during their diagnostic studies prior to any decisions on treatment. Serum samples for basal [FSH] are collected on the third day of the menstrual cycle to assess follicular development. More recently, the use of anti-Müllerian hormone concentration has been incorporated in women’s health mainly since the value is not dependent on the menstrual cycle. This allows for flexibility and scheduling convenience since blood samples can be collected at any patient visit. The observation that [AMH] is a good indicator of blastocysts development during an IVF treatment is not commonly believed since it has not been utilized widely like [FSH]. In the recent years, serum [AMH] has been more widely used as a diagnostic indicator to assess ovarian reserve since the results do not fluctuate with menstrual cycle and provide a stable and reliable value.

Women with elevated basal [FSH] are reported to still achieve reasonable pregnancy rates, but few studies report a correlation of basal [FSH] with blastocysts developmental potential [8–11]. The purpose of the current study is to understand the interactions between the number of mature follicles at trigger, [AMH] and basal [FSH] with blastocyst development in DOR, POI and good prognosis patients. We examined the correlations between diagnostic findings of basal [FSH] and [AMH] and their predictive value in blastocyst development. Diagnostic values of [AMH]/basal [FSH] can be resourceful in determining the optimal simulation protocol and identification of individuals who will not benefit from IVF due to poor prognosis. We hypothesize that those individuals with a plasma [AMH] of ≤1 ng/ml and a basal [FSH] measurement of >8 mIU/ml will have a significantly lower percentage of high-quality blastocysts per treatment compared to individuals that have values considered normal for fertility. The data were analyzed using a decision tree analysis, designed for data sets with numerous data categories, which has emerged as a powerful analytical tool for guiding clinical treatment (See S1 File).

The choice primary outcome measure of success was percentage of high quality blastocysts that developed from the initial number of fertilized ooctyes as superior to the total number of high quality blastocysts per treatment. We opted not to use the total number of high quality blastocysts as the choice outcome measure to ensure that the starting number of fertilized oocytes was factored in to the success. Additionally, success measured as a percentage of initial cohort, allows for the statistical analysis to include dependencies of variables and their multiple interaction weights on the final outcome. A key benefit to this approach is that it will allow the poor prognosis patients to be included in the decision tree to aid in maximizing their success while perturbing the key variables that weigh in on success for good responders by limiting the total medication doses, a cost savings for the patients, and lowering the likelihood of surplus embryos that may impose an emotional and financial burden on the patients in the future.

## Materials and methods

A retrospective analysis was made of frozen embryo transfer treatments performed at a single site, Dallas IVF in Frisco, Texas, referred to as “the Center.” *Dallas IVF* is a registered member of the American Society of Reproductive Medicine and a Society of Assisted Reproductive Technology. The facility is accredited by College of American Pathology (CAP), Clinical Laboratory Improvement Amendments (CLIA) and is registered with the Food and Drug Administration (FDA).

The observational data included IVF treatments over a six-month period from January 1, 2015 to June 31, 2015. The data for treatments included in this study were retrieved using an electronic database management system (eIVF, USA). All data were de-identified to comply with Health Information Portability and Accountability Act (HIPAA). Institutional review board (IRB) approval was not necessary for this study since it was observational only using approved standard of care treatment.

### Study population

All women that underwent IVF treatment were included in the study regardless of age or prognosis. The most recent basal serum [FSH] (samples drawn on days 2–4 of menses) was obtained prior to initiation of treatment. Women were treated with one of the two following protocols:

#### Gonadotropin releasing hormone (GnRH) antagonist

This protocol included the use of medication containing recombinant follicle stimulating hormone (rFSH) to stimulate follicular growth and development followed by addition of GnRH antagonist (i.e., Ganirelix^®^) for the suppression of luteinizing hormone (LH) surge and ovulation by blocking the secretion of pituitary gonadotropins. A medication containing either human chorionic gonadotropin or leuprolide acetate to induce final maturation of oocytes within follicles was administered prior to ovarian retrieval [12, 13].

#### Long agonist protocol

This protocol included the use of a GnRH agonist (i.e., Lupron^®^) to cause pituitary down-regulation during the luteal peak period (7–10 days prior to the start of the menstrual cycle) [14]. After this initial period, medications containing rFSH, with or without LH, were added to promote follicular development while the GnRH agonist was continued to prevent premature luteinization and ovulation. Finally, HCG medication was administered for final oocyte maturity prior to retrieval [13, 15].

### Hormone assays and follicular measurements

Endocrine assays for serum basal FSH, AMH and estradiol concentrations were performed by a reference laboratory (LabCorp, Dallas, TX) using liquid chromatography/tandem mass spectrometry (LC/MS-MS) [16, 17]. Measurements of follicular development were performed at the Center by a sonographer certified by American Registry for Diagnostic Medical Sonography (ARDMS) or a physician with fellowship training in Reproductive Endocrinology and Infertility. Follicle growth was measured in 2 dimensions, calculated and reported as a mean value in millimeters via the ultrasound software.

### Treatment protocol

Women underwent controlled ovarian stimulation using either the down-regulated agonist long protocol or the antagonist protocol, as described above. A baseline transvaginal ultrasound was performed prior to stimulation to ensure ovaries were free of cysts. Both protocols incorporated either recombinant FSH and hMG (human menopausal gonadotropins) or urinary FSH. For the long protocol, down regulation of patients was obtained through leuprolide acetate (Lupron^®^) administration beginning mid-luteal phase. For the antagonist protocol, gonadotropin releasing hormone (GnRH) was administered when the lead follicle reached ≥14mm in diameter or by day 7 of stimulation, at the latest. When a majority of the follicles developed to ≥16mm in diameter in conjunction with appropriately rising serum estradiol levels suggesting oocyte maturation, women were triggered for oocyte retrieval with the administration of 10,000 IUs of human chorionic gonadotropin (HCG) for either protocol or Lupron^®^ trigger with the antagonist protocol. Oocyte aspiration was performed under ultrasound guidance at 36h post-trigger administration. Insemination of oocytes was performed 6 hours post oocyte aspiration. Embryonic culture was performed over a period of six days following transvaginal oocyte aspiration and insemination. Embryos were graded for morphology using the SART grading system [18].

### Definition of outcome

The following definitions apply to the outcomes measured in this study.

#### Minimum [AMH]

Lowest measured anti-Müllerian hormone level (ng/ml) prior to the start of IVF treatment.

#### Maximum [FSH]

Highest basal follicle stimulating hormone level (mIU/ml) measured on menstrual IVF treatment day 3.

#### Mature follicle

A follicle that measures ≥16mm in diameter.

#### [Estradiol] at trigger

Highest measured serum estradiol (E2) level (pg/ml) prior to administration of trigger medication.

#### Total IU medication

Total international units (IUs) of gonadotropins (FSH and LH) administered over the length of the follicular stimulation IVF treatment.

#### Day of trigger

Day of stimulation upon which either HCG or Lupron® were administered.

#### High quality blastocysts

Blastocysts usable for cryopreservation or uterine transfer and reached a minimum grade of B for inner cell mass and B for trophectoderm cells according to the SART grading criteria. *Success*: when ≥40% of fertilized oocytes developed into high-quality blastocysts per treatment.

### Data analysis

The distinct interaction of variables and their influence on IVF treatment outcome were analyzed through the use of a generalized linear model (GLM) employing logistic regression and survival analysis using R software [19, 20]. The generalized linear model includes classical linear regression as a special case which uses the gaussian probability model with an identity link function. The generalized linear model includes a number of probability models that are from the exponential family of probability distributions that are used for modeling the dependent variable. Additionally, there is a transformation for the dependent variable, the so-called *link function*, for the specified probability model of the dependent variable. Final fitting of the model was determined through the use of random forest algorithm, a method which determines classification of variables using a regression approach. Decision tree construction was performed using the evolutionary tree algorithm *Evtree* using R software to maximize discrimination between groups at each node of the decision tree displaying maximal homogeneity in each split rule resulting from subset of the predictors [21]. The evolutionary tree utilized regression and coupled it with a Bayesian information criterion [22]. The accuracy of the prediction model was validated by receiver operating characteristic curve which, in addition to the area under the curve, provides confidence intervals for the assay. Further information on these statistical approaches are provided in the S1 File.

## Results

Data evaluated for individuals who produced >12 mature follicles at trigger was evaluated against the data for individuals who produced ≤12 mature follicles at trigger. The latter group consists of individuals presenting with diminished ovarian reserve (DOR). Total IUs of medication was approximately 1000 IUs more per IVF treatment in the ≤12 mature follicles or DOR group compared to the group producing >12 mature follicles (Table 1). Despite higher medication units, estradiol (E2) at trigger was approximately 1700 pg/ml lower in this group compared to the group of individuals who produced ≤12 mature follicles at trigger, consistent with fewer follicles growing.

**Table 1:** Comparison of treatments reflected by number of mature follicles at trigger.

	> 12 mature follicles	≤12 mature follicles
Parameter	n = 61	n = 45
Female age (years)	34.4 ± 0.6	35.9 ± 0.7
Male partner age (years)	37.9 ± 0.9	38.6 ± 0.9
Total IUs of gonadotropins/IVF treatment	3070.1 ± 191.0	4110.8 ± 138.6
Estradiol level at trigger (pg/ml)	4547.8 ± 257.6	2821.6 ± 227.9
Max basal FSH (mIU/ml)	6.6 ± 0.4	7.8 ± 0.2
Min AMH (ng/ml)	5.4 ± 0.5	3.2 ± 0.7
# Oocytes retrieved	22.9 ± 1.3	14.9 ± 1.4
# Oocytes normally fertilized	14.2 ± 0.9	8.8 ± 1.0
# High quality blasts	5.4 ± 0.4	3.3 ± 0.4
%High quality blasts	38.9 ± 3.5	38.9 ± 2.4

Mean values are reported ± std. error

**Fig 1:**
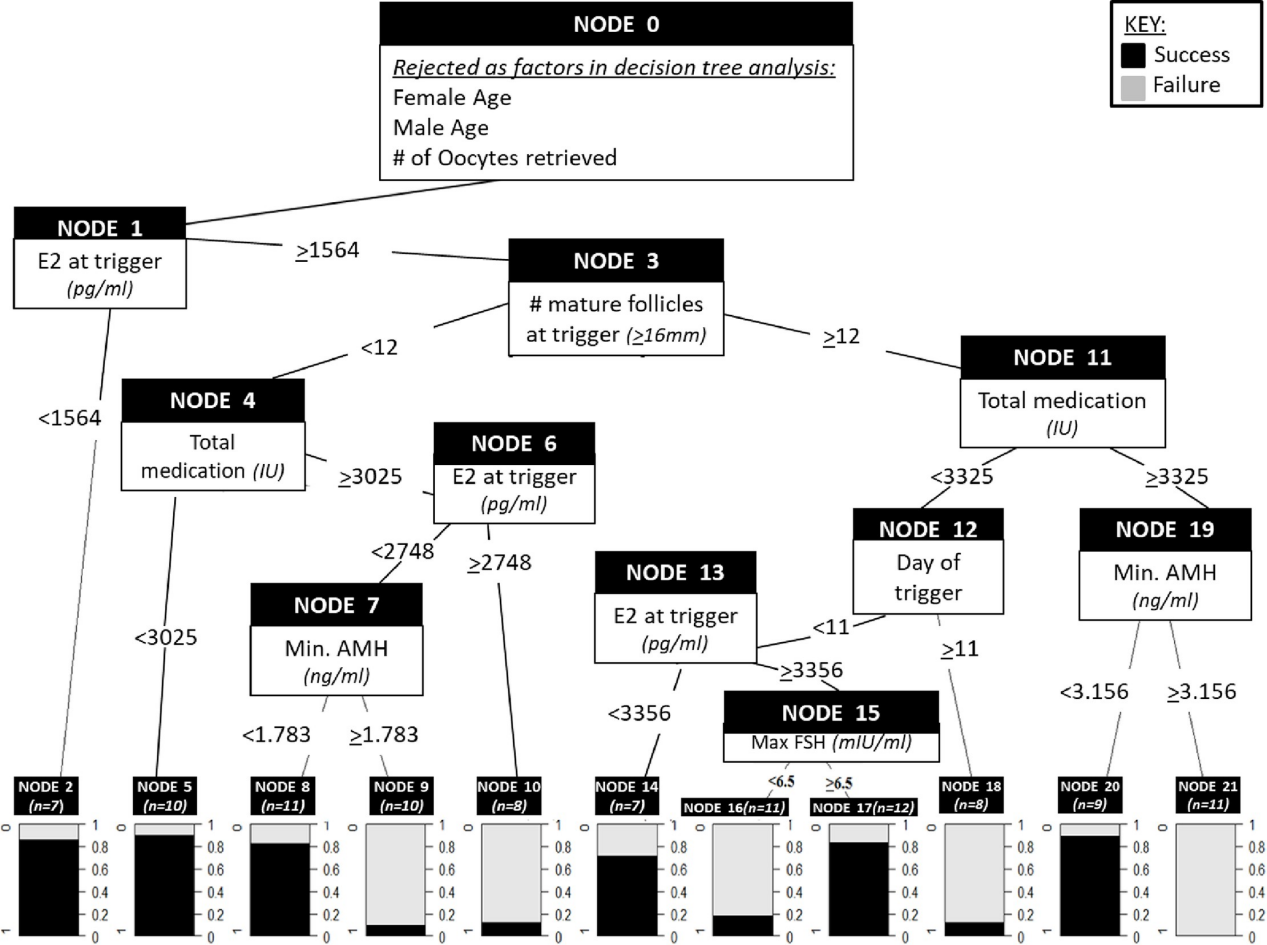
Decision tree analysis (Evtree) for blastocyst optimization. The predictive modeling analysis determined that [E2] at trigger <1584 pg/ml resulted in 80% probability of >40% blastocyst development success (n = 7, node 2). Female age, male age and number of oocytes retrieved were not significant variables (Node 0). [E2] at trigger significantly impacted outcome when [E2] was >1584 pg/ml, based on the number of mature follicles at trigger (<12 vs. >12) (node 2). The branch originating from the left of node 3 (nodes 4–10) demonstrates outcomes of treatments with < 12 mature follicles at trigger. When <3025 IU gonadotropin was administered in this branch, a 90% blastocyst development success occurred (n = 10, node 5). Total IU medication >3025 IU for with an [E2] at trigger of <2748 pg/ml and minimum [AMH] <1.783 ng/ml for 80% blastocyst development success (n = 11, node 8), otherwise success was poor (nodes 9, 10). The branch on the right of node 2 (nodes 11–21) depicts outcomes for treatments with >12 mature follicles at trigger. When [E2] at trigger >1584 pg/ml, success was significantly improved by administering <3025 total IU medication with and trigger by day 10 (node 13). [E2] <3056 pg/ml resulted in 70% (n = 7, node 14). [E2] >3056 pg/ml when maximum [FSH] was >6.5 mIU/m1 resulted in 85% success (n = 12, node 17). Success was > 90% success seen with [E2] >1564pg/ml, >12 mature follicles at trigger, >3325 IU medication and minimum [AMH] of <3.156 ng/ml (n 9, node 20).

The generalized linear model determined minimum [AMH], total IU medication, number of mature follicles at trigger and E2 at trigger to be significant contributors towards optimal blastocyst development. A median of 40% fertilized oocytes developed into high-quality blastocysts from all treatments included in this study (Table 1). *Evtree* analysis was employed and discriminated the outcome per treatment over the median of all treatments to obtain optimization of blastocyst development. The analysis found treatments in which a [E2] of <1584 pg/ml at trigger resulted in 80% probability of success (≥40% blastocyst development) (Fig 1, node 2). The combination of [E2] at trigger ≥1564 pg/ml, the number of mature follicles at trigger was <12, and total IU medication <3025 IU, treatments resulted in 90% success (Fig 1, node 5). Treatments in which total IU medication was >3025 IU, [E2] at trigger was <2748 pg/ml and minimum [AMH] was <1.783 ng/ml, then 80% success was observed (Fig 1, node 8). Importantly, higher minimum [AMH] (>1.783 ng/ml) and [E2] at trigger resulted in <15% success (nodes 9, n = 10). Yet, when ≥12 mature follicles were present, <3325 IU of medication administered, trigger administered by day 10 and [E2] at trigger <3356 pg/ml, treatment success was 70% (Fig 1, node 14). Treatments in which the maximum basal [FSH] was ≥6.5 mIU/ml and [E2] at trigger was >3056 pg/ml yielded 85% success (Fig 1, node 17). In contrast, with the similar E2 response and medications administered in individuals whose basal [FSH] was <6.5 mIU/ml, treatment success declined to ~20% (Fig 1, node 16).

**Fig 2:**
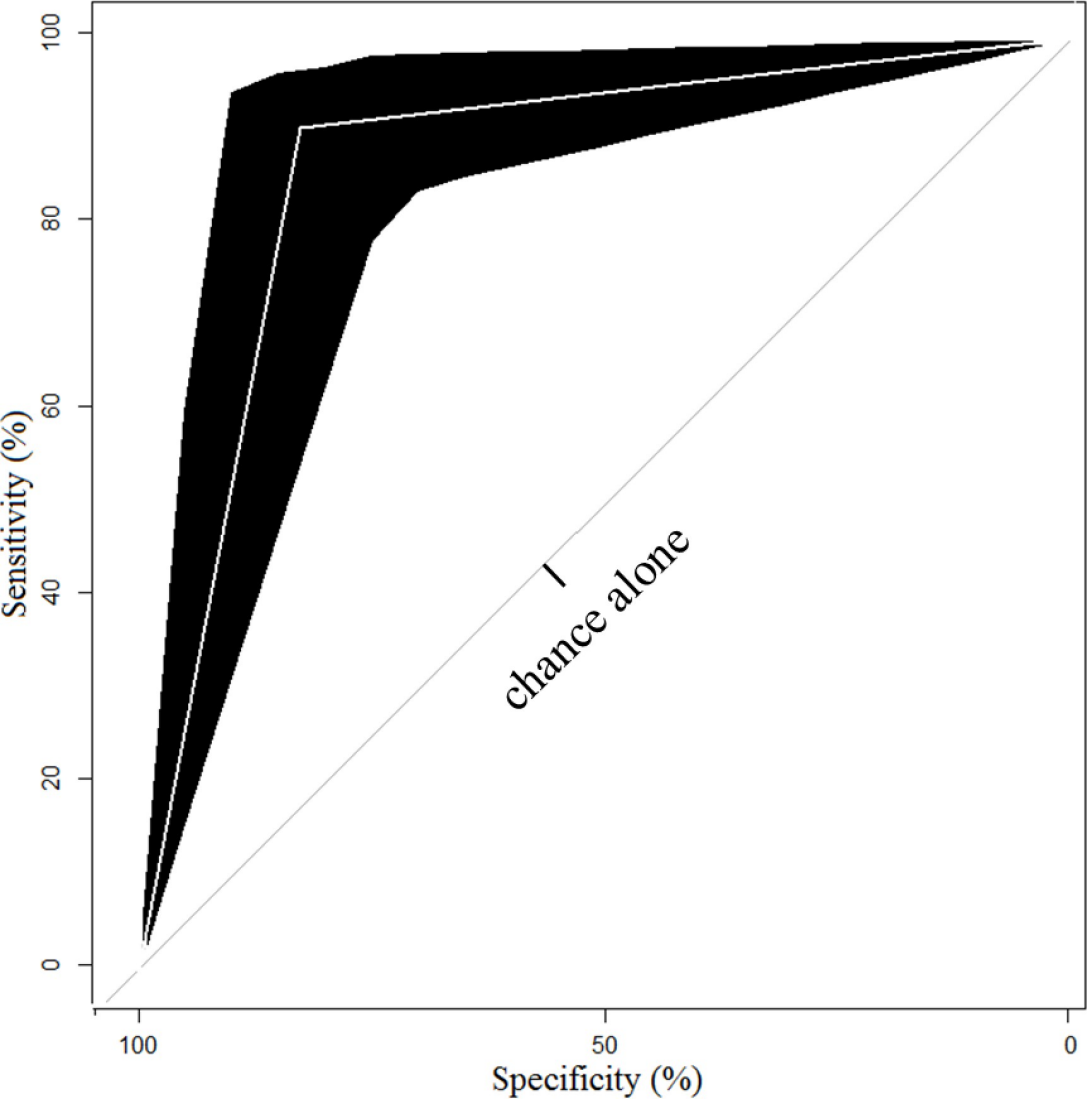
Receiver operating characteristic curve of the blastocyst optimization model. The accuracy of the decision tree was calculated at 86.5% with an error rate of 13.5%. The area under the ROC curve (Robin et al. 2011), which is a plot of the true positive rate (sensitivity) against the false positive rate (specificity), is approximately 0.87 with confidence intervals of 0.7996–0.9312. This indicates that any final node on the model will yield 29% greater success than chance alone at the lowest predictive accuracy and 43% greater success than chance alone at its highest predictive accuracy. This is added validation of the predictive model in the current study.

Success was over 90% when [E2] at trigger was ≥1564 pg/ml, ≥12 mature follicles at trigger, >3325 IU medication and minimum [AMH] of <3.156 ng/ml (Fig 1, node 20). The accuracy of the decision tree is 86.5% with an error rate of 13.5%. The likelihood of an outcome being other than what is predicted by the model, or the chance that the model is incorrect, is just 13.5%. The receiver operating characteristic (ROC) curve [23] area is 0.87 (Fig 2). Confidence intervals (CI) of 0.7996–0.9312 indicate that any final node on the model will yield 29% greater success than chance alone at the lowest predictive accuracy and 43% greater success than chance alone at its highest predictive accuracy.

## Discussion

Basal [FSH] and minimum [AMH] are employed as clinical indicators to determine IVF treatment prognosis for pregnancy [2, 3, 9]. Yet, there remains a gap in the crucial understanding of how these hormone concentrations correlate with blastocyst developmental potential—the single performance indicator that has the highest impact on pregnancy outcome, as supported by our decision tree results. The primary reason for this gap is due to the widely employed gold standard of “live birth” for measuring treatment success, which has limited research focusing on other alternative outcome measures that place a large premium on live birth. It is therefore pertinent to bridge this gap with the goal of ultimately improving live birth by using the decision tree results of the current study in future treatment decisions to maximize the number of high-quality blastocyst production per treatment. The current results indicate that decision tree application will result in fewer treatment attempts to a single successful pregnancy. The wider intention of using the decision tree model is to have fewer treatments per individual and result in greater than one opportunity for pregnancy given more available high-quality blastocysts per treatment.

### Outcomes in individuals with high-ovarian reserve (≥12 mature follicles at trigger)

Remarkably, our analysis found that individuals who present with higher ovarian reserve and traditionally are considered good prognosis ([AMH] >3.2 ng/ml) actually experience lower success for blastocysts developmental rate compared to individuals with low ovarian reserve. We have observed in this study that greater numbers of follicles developing simultaneously may demonstrate decelerated growth per follicle on ovarian ultrasound, since the gonadotropin dose is shared amongst follicles. This depicts the magnitude of physiological role and differential response of follicular development in high response individuals, which contributes significantly to outcome. To combat the lag in follicular development in the high-responder group, gonadotropin doses are increased by clinicians to provide sufficient stimulation for follicles and promote growth. According to our results, increasing total IU of gonadotropins threatens the outcome by substantially reducing overall blastocyst developmental rate. In support, there is a strong correlation between the rate of follicle development and total IU of gonadotropins administered during treatment, which greatly diminishes success (0/11 successful treatments) when the individual is over-exposed to gonadotropins (Fig 1, node 21). A decision tree analysis can be used to determine an individualized treatment option for patients with high ovarian reserve. We recommend that ovarian response can be significantly controlled by extended use of oral contraceptive pills (OCPs) prior to initiating treatment to aid in ovarian suppression. Given a compelling outcome of 0% success in this arm of the analysis, we also strongly recommend limiting the total medication units for individuals presenting with an [AMH] of ≥3.156 ng/ml to <3325 IU per treatment. The combination of extended OCPs and limiting gonadotropic stimulation medication may result in a more even recruitment of fewer follicles, along with a better controlled ovarian stimulation and E2 response for this group. All of these approaches are likely to increase success in blastocyst optimization and ultimately, pregnancy.

### Outcomes in individuals with low-ovarian reserve (<12 mature follicles at trigger)

In contrast to good prognosis individuals who present with high ovarian reserve, individuals which present with low ovarian reserve have elevated basal [FSH] which is associated with a reduced overall live birth rate [24, 25]. These individuals are generally poor responders with poor prognosis in part, due to diminished ovarian reserve (DOR). In contrast, our results indicate treatments with <12 mature follicles at trigger (Fig 2, node 3), is associated with high success in blastocysts developmental rate when gonadotropins are <3025 total IUs and estradiol at trigger is under 2750 pg/ml. The association of these outcomes with the *in vivo* physiology of oocytes throughout stimulation is key in determining a successful stimulation dosing protocol. Future studies are needed to investigate the intra-follicular and the intra-cellular mechanisms which lead to the inverse relationship of blastocysts development and total gonadotropin doses in good responders in contrast to poor responders.

We reject the hypothesis that women with an [AMH] of ≤1 ng/ml and a basal [FSH] >8 mIU/ml will have significantly lower percentage of high-quality blastocysts compared to women that have values considered normal for fertility. Instead, the current study indicates that individuals with elevated [FSH] had ≥12 mature follicles at trigger and, importantly, over 80% of treatments were successful when total gonadotropin medication doses are <3325 IU. Thus, basal FSH concentrations ≥6.5 mIU/ml does not correlate with lower blastocysts development rate success (Table 1), as is commonly believed. Instead, we found that [AMH] is a more reliable indicator of blastocyst developmental potential compared to basal [FSH]. Patients presenting with [AMH] <1.783 ng/ml generally have <12 mature follicles at trigger and over 80% of treatments were successful when estradiol at trigger was <2750 pg/ml even when total IU of medication exceeded 3000 IU.

In clinical practice, the E2 response on day of trigger can be difficult to predict. The decision tree (Fig 1), can be applied to treatments by using the diagnostic data available and working from the final success node backwards depending on the expected number of mature follicles at trigger based on the patients antral follicle counts prior to stimulation medication start. The decision tree is a powerful tool in that that the results outlined have taken responses from 106 cycles and created groups based on statistical weights of variables included. The groups reflect significant categorization of variables in treatments which lead to predicted success in outcome. Depending on the patients’ baseline characteristics, only certain arms or sections of the decision tree will be applicable to each treatment. For example, in Fig 1, [AMH] and [FSH] values can be plugged in the decision tree in conjunction with patient’s antral follicle counts substituted as number of mature follicles at trigger. With these values plugged in, the clinician can work backwards form the final outcome node predicting the highest success in the specific section of the tree to find the limits on total medication dose and day of trigger.

E2 on trigger day is not more critical than the [FSH] and [AMH] level of patients. On the right side of figure, under the section “≥12”, at node 11 (total IU of medication) there is a significant difference observed in outcome. This arm of the decision tree is for those patients who present with high ovarian reserve, are expected to have ≥12 mature follicles at trigger based on their antral follicle count at the beginning of the treatment and have administered gonadotropin doses of ≥3325 IU. Patient’s [AMH] value should be considered prior to dose setting. If the patient presents with <3.156 [AMH] (node 19), then the gonadotropin dose of ≥3325 IU (node 11) will result in 89% likelihood of success (node 20) versus if the patient presents with [AMH] ≥3.156 (node 21, 0% success). In this case, the gonadotropin medication dose of ≥3325 IUs is too high and should be reevaluated. Significance in outcome or variability in success, is not observed in this example (≥12 mature follicles at trigger, [AMH] of <3.15ng/ml, final node 20) if the maximum medication dose is = 3325 or a higher value. In this case, for the financial benefit of the patient, the dose may be prescribed at 3325IU and administered equally over the total number of days of stimulation.

We confirmed that patients with DOR or poor prognosis can exhibit a high rate of good quality blastocyst development per IVF treatment. This outcome positively correlates with increasing levels of gonadotropins, which is contrary to individuals with a higher number of mature follicles at trigger.

### Recommended stimulation protocols

Based on the analysis of our predictive model, the following protocols are suggested for patients with particular characteristics to promote the maximum number of high-quality blastocysts development. These protocols should be validated against the decision tree by following the pathways of decision at each patient visit during stimulation. Adjustments to the gonadotropin medication doses should be made dependent on patient E2 response. Total IU of medication should be monitored to not exceed the recommendation of the decision tree.

***>12 mature follicles expected at trigger***

- Patient criteria:
  ■ [AMH]: (>1.8 but <3.2 ng/ml)
  ■ [FSH]: >6.5 mIU/ml
  ■ Antral follicle count ≥12.
- Protocol:
  ■ Consider extended OCPs, 21 days recommended.
  ■ Antagonist using Ganirelix® or Agonist suppression using Lupron®.
  ■ Combined gonadotropin dose of 300 IU/day, ≤10 days with the goal of maintaining E2 <3350 pg/ml.
- Trigger:
  ■ With hCG (10,000 Units) or leuprolide acetate on day 8–10, with E2 ≤3350 pg/ml.

***<12 mature follicles expected at trigger***

- Patient criteria:
  ■ [AMH]: (<1.8 ng/ml).
  ■ Antral follicle count <12.
- Protocol:
  ■ Antagonist using Ganirelix.
  ■ Combined gonadotropin dose of 275 IU/day, ≤11 days with the goal of maintaining E2 <2748 pg/ml.
- Trigger with hCG (10,000 Units) on day 8–11, with E2 ≤2748 pg/ml.

## Conclusion

AMH concentration is a more dependable indicator of blastocyst developmental potential in IVF treatments in comparison to basal [FSH]. Women that present with infertility due to DOR or women who undergo treatments with high [AMH], if managed appropriately, can produce more usable blastocysts per IVF treatment, thus increasing the potential for a successful pregnancy. Implementation of our decision tree results will reduce total treatment costs and lower emotional burden. The physiological response to stimulation medication produces significantly different outcomes for good responders compared to poor responders for IVF. It is recommended that reproductive professionals implement and apply concrete and well supported treatment options. In this regard, the decision tree approach offered by the current study will lead to improved outcomes and lower stressors associated with infertility treatments. Furthermore, it is recommended that reproductive biologists and clinicians broaden their understanding of “success” and define it as multiple pregnancy attempts per treatment as a result of optimal blastocyst development.

## Supporting information

S1 File (DOCX)

S1 Data (CSV)

## Glossary and terms

AMH: Anti-Müllerian Hormone
DOR: Diminished ovarian reserve
E2: Estradiol
ET: Embryo Transfer
FSH: Follicle stimulating hormone
GLM: Generalized linear model
GnRH: Gonadotropin releasing hormone
HCG: Human chorionic gonadotropin
HIPAA: Health Information Portability and Accountability Act
hMG: Human menopausal gonadotropin
IU: International units
IVF: *In Vitro* Fertilization
POI: Preliminary ovarian insufficiency
