# Supplementary material for: Maternal serum concentration of anti-Müllerian hormone is a better predictor than basal follicle stimulating hormone of successful blastocysts development during IVF treatment
Source: PLoS One. 2020 Oct 12;15(10):e0239779. doi: 10.1371/journal.pone.0239779 (PMC7549822; doi:10.1371/journal.pone.0239779)
Supplement: S1 File — (DOCX) [file pone.0239779.s001.docx]

**Statistical Methods**

Machine learning derives from computer science and artificial intelligence with systems that learn from the experimental data, as compared to analysis based on explicitly programmed instructions (1). Machine learning provides an impressive processing power using programs to make successful predictions based on highly complex data sets. The process has evolved through fields like computer science and physics and are often referred to as “machine learning”, “artificial intelligence”, “data mining”, “predictive analysis”, etc. (2). This form of artificial intelligence is programmed to “learn” as the characteristics of the data set change. Given the power of analysis that predictive modeling provides for datasets involving 10 or more variables, we chose analysis methods using the *Evtree* algorithm.

***Evtree Analysis***

Evtree, short for evolutionary tree, is an algorithm for predictive modeling based on a genetic and global optimization method (Grubinger et al., 2014). Inspired by Darwinian evolution, the algorithm employs concepts such as inheritance, natural selection and mutation to calculate a “decision tree”. Population-based trees are simultaneously fitted by *variation operators* called *mutation* and *crossover* which merges various solutions. Ultimately, the “survivor” selection is an evolutionary process in which the quality of the population is increased over time (4).

The basic unit and foundation of predictive modeling and the Evtree-based analysis is a “tree” with one “main node”, which is the trunk from which all possible combinations stem. This tree then splits into smaller branches with the split at each branch referred to as a “node”, and the “leaves” of each branch representing a homogeneous sub-group (figure S1). This tree-based regression approach creates thousands of trees, each with its own, unique dataset. The variables are weighted for importance using the mean decrease Gini index, a coefficient to measure the contribution of each variables to the homogeneity of the nodes and leaves in the final model (5,6). For each split, the Gini index coefficient of the resulting node is calculated, and a comparison is made to the original node ultimately producing a weight of each variable in the final decision tree or predictive model. The Evtree algorithm randomly selects predictions at each split, starting with the most weighted variable at the top, creating “nodes”. Each node then produces two subgroups called “leaves”. The algorithm learns and tunes the tree over many thousands of iterations using the similarities prevalent in each generated tree generated and produces a final optimized tree with rules for the split. The split rules are created by maximally discriminating between the two subgroups (leaves) providing the split (node) but maintaining the most homogeneity within each of the independent subgroups.

The analysis of the current study used Bayesian Information Criterion (BIC) to fit the survivor model (7). Initialization of the analysis was conducted by a valid, randomly generated root node. From this point, iterations were only selected once to be altered by the variation operators to give rise to the parent nodes. The evaluation function of the algorithm formulates, through statistical and mathematical computations, requirements for the population adaption. In this approach each parent solution competes for its place in the population with the offspring. The split rules are determined for the offspring population to be discriminate from the parent population but mutated from the sibling population in terms of nodes on the tree. The algorithm identifies the parent population as independent variables and the offspring population to have dependence on the parent population. The offspring population is then evaluated against other variables in this tier, referred to as the sibling population. The algorithm identifies the differences in each variable, their dependence on the variables in branches above them and their impact on variables in the branches below. Finally, termination of the analysis is reached when the top 5% of the generated trees are stabilized for a minimum of 100 iterations. Evtree does not perturb variables due the evolutionary nature of the analysis, nor does it use bootstrap re-sampling or cross validation like random forests algorithms. Rather, the evtree package as deployed in R software implements an evolutionary based algorithm used to learn the classification of global optimality of a dataset.

## *Receiver Operating Characteristic Curves*

A receiver operating characteristic (ROC) curve is a tool for evaluating classifiers in applications utilizing datasets for bioinformatics (8). A ROC takes the form of a graphical plot showing the strength of the diagnostic ability of a binary classification (positive/negative) system as the threshold of this system’s discrimination varies (9). The curve is created by plotting the true positive rate of a model against the false positive rate over varying thresholds. The true positive rate is known as ‘sensitivity’ or the probability of detection in machine learning (10). Similarly, the false positive rate is known as ‘specificity’ and can be calculated using the formula: (1-specificity) = False Positive / (False Positive + True Negative), expressed as a percentage. In a prediction model using the probability distributions of detection rate and specificity, the ROC curve is generated by plotting the area under the curve as the probability of distribution up to the discrimination threshold of the detection probability on the x-axis and the distribution function of sensitivity on the y-axis.

The ROC curve maps output in classes. In a binary classification system, there are two outcomes: positive and negative. If the prediction model predicts an actual positive value as positive, it is plotted in the top left quadrant. However, actual negative observations which are predicted as positive by the model are plotted in the top right quadrant and are false positive, also known as type I errors. Actual positive values predicted as negative are plotted in the bottom left quadrant, a type II error. Finally, the bottom right quadrant represents actual negative values predicted to be negative by the model. Figure S2 is an overlap of the binary classification table with a ROC curve. The area under the curve is referred to as the trapezoidal area under the curve to the 45˚ diagonal line stretching from the bottom left (100% specificity, 0% sensitivity) to the top right (0% specificity, 100% sensitivity). To summarize, the farther in the top left quadrant the ROC curve stretches, the stronger the predictability power of the model resulting in higher chances of the model being accurate when it predicts an actual positive condition as positive (2). As an example, accuracy of the predictive model at 50% states the likelihood that the prediction of the final outcome is correct is equivalent to chance alone. The perfect prediction model that predicts final outcome correctly each time will have an accuracy of 100%. Our predictive model had an accuracy of is 86.5%, meaning the use of the model will result in success 36.5% more often than not using the model and relying on chance alone.

The ROC curve analysis is performed to determine accuracy of the entire final prediction model as a whole, validating the accuracy of any final node/leaf on the tree. An accuracy value of 50% indicates that the predictive model has little meaning - the prediction is just as likely to emerge by chance alone. Any value higher than 50% will give you higher rate of success than chance alone. The results of the present study were >85% accurate. This means that by using the predictive model, we have a 35% higher likelihood of success (at 85%), than chance alone (50%).

ROC curves are calculated to consider all factors acting simultaneously and instantaneously on outcome as the discrimination threshold varies of the final prediction model. Therefore, ROC curves are a useful tool in validating prediction models.

# References

1. Baştanlar Y, Ozuysal M. Introduction to machine learning. Methods Mol Biol Clifton NJ. 2014;1107:105–28.

2. Kuhn M, Johnson K. Applied Predictive Modeling [Internet]. New York, NY: Springer New York; 2013 [cited 2017 May 18]. Available from: http://link.springer.com/10.1007/978-1-4614-6849-3

3. Grubinger, Thomas, , Achim, Pfeifer, Karl-Peter. evtree: Evolutionary Learning of Globally Optimal Classifcation and Regression Trees in R. J Stat Softw. 2014 Sep;61(1):29.

4. Eiben AE, Smith JE. Introduction to Evolutionary Computing [Internet]. New York, NY: Springer-Verlag; 2007 [cited 2017 Oct 20]. Available from: //www.springer.com/us/book/9783642072857

5. Menze BH, Kelm BM, Masuch R, Himmelreich U, Bachert P, Petrich W, et al. A comparison of random forest and its Gini importance with standard chemometric methods for the feature selection and classification of spectral data. BMC Bioinformatics. 2009 Jul 10;10:213.

6. Handyside AH, Montag M, Magli MC, Repping S, Harper J, Schmutzler A, et al. Multiple meiotic errors caused by predivision of chromatids in women of advanced maternal age undergoing in vitro fertilisation. Eur J Hum Genet EJHG. 2012 Jul;20(7):742–7.

7. Volinsky CT, Raftery AE. Bayesian information criterion for censored survival models. Biometrics. 2000 Mar;56(1):256–62.

8. Robin X, Turck N, Hainard A, Tiberti N, Lisacek F, Sanchez J-C, et al. pROC: an open-source package for R and S+ to analyze and compare ROC curves. BMC Bioinformatics. 2011;12(1):77.

9. Powers DM. Evaluation: from precision, recall and F-measure to ROC, informedness, markedness and correlation. 2011;

10. Froud R, Abel G. Using ROC curves to choose minimally important change thresholds when sensitivity and specificity are valued equally: the forgotten lesson of pythagoras. theoretical considerations and an example application of change in health status. PloS One. 2014;9(12):e114468.


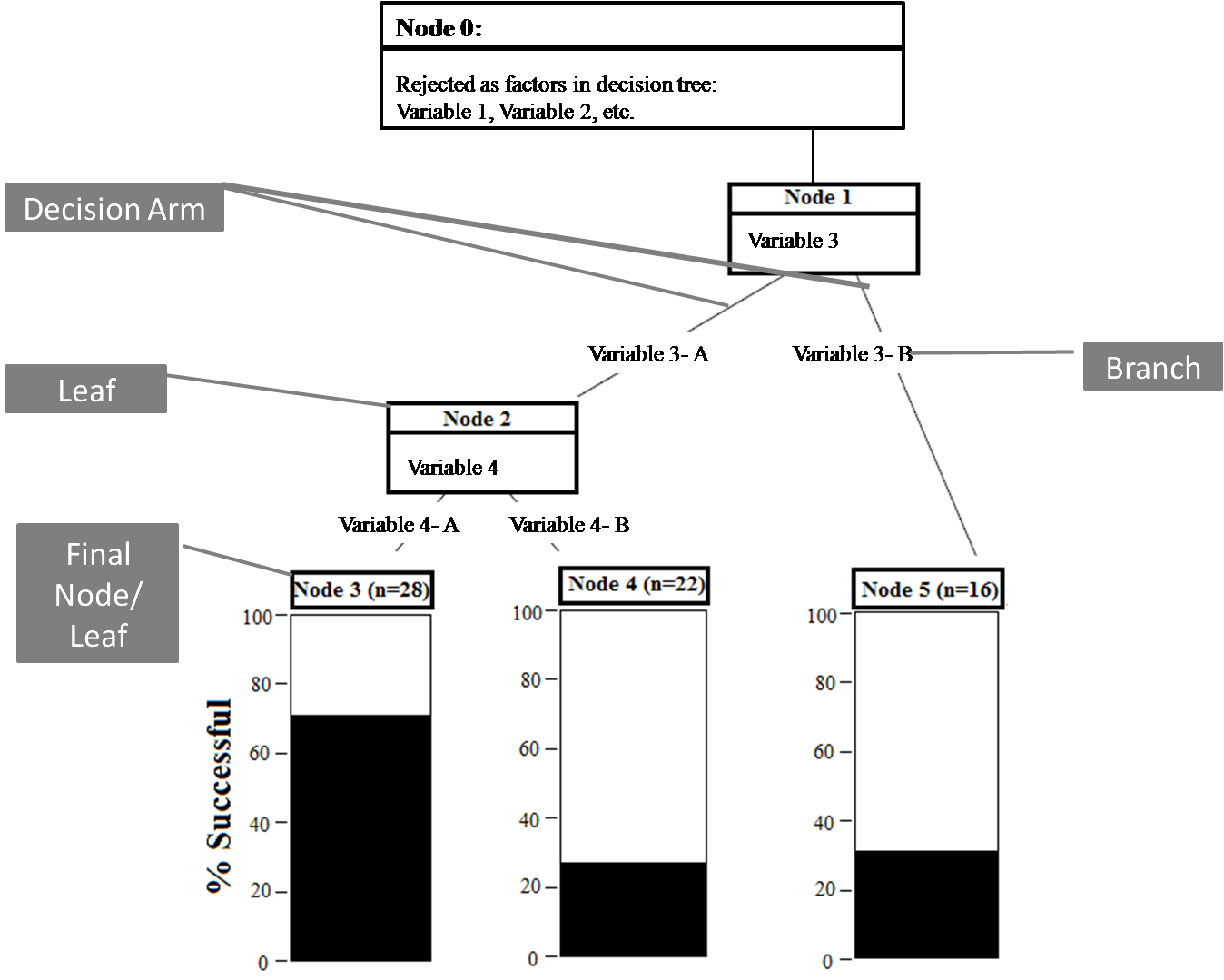


**Figure S1** Decision tree. This figure is representative of a predictive modeling decision tree. The gray boxes indicate the terminology associated with the various parts of the model.


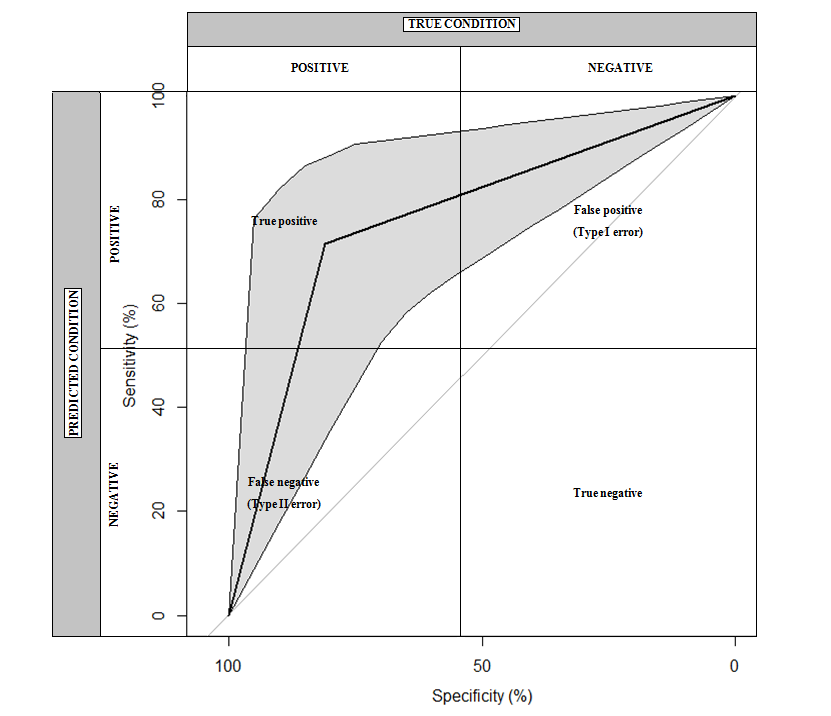


## Figure S2- Receiver operating curve

A sample curve is superimposed on a prediction table. The 45˚ line demonstrates the role of chance for any predicted condition to result in the true condition. The gray areas of the curve represent confidence intervals where the lowest predictive success and the highest predictive success of the model can be calculated.
